# Disparities in Diet Quality and Food Security Across Ethnic-Immigration Status and US Nativity

**DOI:** 10.1007/s40615-025-02425-6

**Published:** 2025-04-16

**Authors:** Abiodun T. Atoloye, Francis Tayie, Sherif O. Olasoji

**Affiliations:** 1 Department of Nutrition, Dietetics, and Food Sciences, Utah State University, 8700 Old Main Hill, Logan, UT 84322 - 8700 USA; 2 Department of Allied Health, & Sport Sciences, Kinesiology, Southeast Missouri State University, Cape Girardeau, MO USA

**Keywords:** Ethnic-immigration status, US nativity, Healthy eating index, Food security, Diet quality, Cross-cultural learning

## Abstract

This study examines separate relationships between ethnic-immigration status, length of residence (US nativity), dietary quality, and food security status among US adults. Data from the National Health and Nutrition Examination Survey between 2017 and 2020 were used. The study sample included 6933 adults aged 18 and above. Food security status was categorized as either food secure or food insecure. Dietary quality was assessed using the Healthy Eating Index (HEI-2015) component scores. The ethnic-immigration status comprised US-born and immigrant groups, including Whites, Asians, Blacks, Hispanics, and multi-racial group. The US nativity was categorized as native, less than 5 years, 5–14 years, 15–30 years, and above 30 years of residency. Associations between ethnic-immigration status, US nativity, and food security were analyzed using logistic regression. Their associations with dietary quality used linear regression. The results showed that US-born Hispanics, multi-racial group, and immigrant Hispanics had about twice the odds of being at a risk of food insecurity compared to their US-born White counterparts, while US-born Blacks had about one and half odds. Food insecurity was higher among respondents with less than 5 years and over 30 years of residency in the USA, with odds slightly less than two. Immigrants had better overall dietary quality than US-born Whites and natives (*p*-values < 0.001). The study highlights the importance of nutrition interventions that consistently promote equitable access to affordable and nutritious foods while fostering the exchange of valuable dietary practices among groups and encouraging cross-cultural learning to improve overall health.

## Introduction

The Nutrition Health Disparities Framework highlights socio-cultural environment which encompasses acculturation, cultural identity, and language proficiency, as one of the multiple determinants that influence nutrition-related health outcomes [1]. The process of acculturation is a complex and multidimensional process involving the acquisition of a new language, adaptation of cultural practices, social interactions, values, beliefs, behaviors, and identity [3]. This change can influence economic stability, social networks, and access to resources. As individuals and families adapt to a new cultural environment, they may experience changes in their dietary habits, food preferences, and access to familiar foods [2]. These changes can either mitigate or exacerbate nutrition-related disparities. Factors such as immigration status and race/ethnicity are crucial demographic variables that provide insight to language proficiency, immigration history, cultural heritage, and norm. These factors may shape how an individual navigate and interact with the food environment, leading to differences in food insecurity outcomes and contributing to disparities in health outcomes [4].

Previous research on immigrant health indicates that United States (US) immigrants often arrive with relatively good health despite having a lower socio-economic status [5–7]. However, this initial health status tends to diminish with longer residency in the country. This suggests that nutrition-related outcomes, as well as the social and physical environments, may contribute to the overall health of immigrants in distinct ways compared to the general population. Moreover, individuals may acculturate differently across various dimensions, leading to varied outcomes [3]. However, acculturation theory often does not consider how overlapping cultural/social identities, such as race, ethnicity, and gender influence the acculturation process [8, 9], leading to the misrepresentation of experience of individuals from diverse cultural background. Therefore, it is crucial to understand how cultural and social identities (e.g., ethnicity/race) shape people experience as they adopt the host culture. Examining socio-cultural environmental factors like ethnicity, race, length of residency, or nativity can offer a deeper understanding of the acculturation process and its influence on nutrition-related outcomes such as food insecurity and diet quality. It is also essential in developing culturally responsive policies and programs.

Previous population-based studies that have examined differences in food security have used migration history as an acculturation proxy to explore these relationships [10–12]. For instance, one reported that being a non-citizen is a risk factor for food insecurity [10, 11]. In contrast, another study reported that food security issues are most pronounced in new immigrants, lessen after 5–14 years in the US, and then increase again after 15 years [12]. Four other studies examined food security and dietary quality among different ethnic/racial groups [13–16]. One of these studies, which focused on a subsample of individuals with non-alcoholic fatty liver disease, reported that White individuals had lower dietary quality compared to other ethnic/racial groups [14]. Another study reported that food insecurity was linked with lower diet quality primarily among non-Hispanic Whites, Asians, and adults classified as “other” [15]. Another study suggests that Black and Hispanics immigrants may experience lower food security compared to their native counterparts, while Asian immigrants from China and India have higher food security levels [16]. The last study examined food insecurity among ethnic and racial groups by nativity and language use and reported that Blacks and Hispanics are significantly more food insecure than both foreign- and native-born Whites regardless of nativity status [13]. These studies suggest that migration status is a risk factor for food insecurity, with long-term immigrants facing renewed food security challenges. Additionally, Black, Hispanic, and Asian populations experience disproportionately higher rates of food insecurity compared to White populations. However, the mixed role of nativity status further suggests the potential influence of systemic factors in driving these disparities. While the differences in food insecurity by various dimensions of social-cultural environmental factors (migration history/nativity/county of birth, language use, race/ethnicity) have been well researched previously, to the best of our knowledge, few studies have explicitly examined the differences in diet quality or food security by subgroups that shared characteristics such as ethnic background, nativity, and length of residency.

The mixed finding on the role of nativity (whether someone is foreign-born or native-born)—an acculturation proxy—highlights the need for further research. Given that acculturation does not occur in isolation but may be shaped by immigration status, racial, or ethnic background, understanding these factors through the lens of intersectionality will provide more comprehensive perspective on how these intersecting identities influence individuals’ access to healthy food and overall diet quality. This approach is essential for designing targeted interventions that address the unique challenges and needs of both immigrant and native communities, ultimately promoting better health outcomes. The current study fills this gap by using a nationally representative dataset to examine the associations of ethnic-immigration status (a combined consideration of an individual’s ethnic background and nativity) and US nativity (native vs foreign-born status, with foreign-born status categorized by length of residency levels) with food insecurity and diet quality. First, we compare food insecurity risk across different ethnic-immigration status and nativity; and second, we compare diet quality across different ethnic-immigration status and nativity.

## Methods

### Study Population

This study utilizes existing data obtained from adults aged 18 years and older who participated in the pre-pandemic 2017–2020 National Health and Nutrition Examination Survey (NHANES) [17]. The NHANES is conducted by the National Center for Health Statistics (NCHS) of the Centers for Disease Control. The NHANES utilizes a stratified, complex multi-stage probability cluster sampling design to create a population sample representative of the non-institutionalized US population. The NHANES complex sampling design requires the use of sampling weights to produce nationally representative outcomes. Oversampling of selected population groups was conducted to increase the reliability of estimates of nutrition indicators.

For this secondary analysis, 6933 participants were included after satisfying the inclusion criteria. With a percentage missingness of 28%, participants were excluded if the following variables were missing:

- Food security (*n* = 736).
- Nativity (*n* = 120).
- Diet quality (*n* = 1298).
- Body mass index (BMI) (*n* = 91).
- Educational level (*n* = 332).

**Fig. 1:**
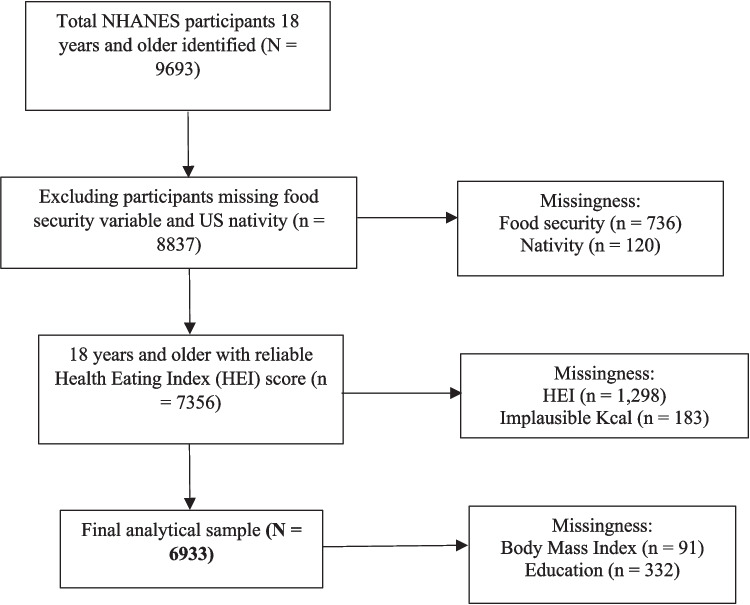
CONSORT diagram for adult participants aged 18 years and older in NHANES 2017–2020 for examining disparities in diet quality and food security across ethnic-immigration status and US nativity

In addition, participants were excluded if they reported extreme caloric intake (< 500 or > 5000 kilocalories per day, *n* = 183) as this indicates potential misreporting of dietary intake. Figure 1 provides more details.

The NHANES protocols were reviewed and approved by the National Center for Health Statistics’ ethics review board and all participants provided written informed consent (Protocol #2011-17 and #2018-01 ) [18]. The NHANES is conducted in accordance with the Code of Ethics stated in the Declaration of Helsinki on experiments involving human subjects. The NHANES datasets used for this study had been deidentified and released for public use. Thus, the secondary analysis of the publicly available NHANES datasets was considered exempt from further review by the Utah State University Institutional Review Board.

### Measures

#### Socio-cultural Environmental Factors

From the dataset, the socio-cultural environmental variables of interest, namely ethnicity, country of birth, and length of residency in the US, were used to generate the main exposures of interest (US nativity and ethnic-immigration status) using the categorization approach to construct intersectionality variables [19]. Country of birth is a common variable for both exposure of interest which indicate whether the participant is foreign-born or native-born, similar to nativity explained above. Although an interaction term approach would be suitable for ethnicity and country of birth, the categorization approach was chosen to eliminate multicollinearity between country of birth and length of residency and maintain consistency across both exposures of interest. The categorization approach identifies subgroups based on their shared characteristics into a single variable. This involved combining ethnicity and country of birth as indicator of participants’ ethnic-immigration status [20]. Based on the major racial and ethnic groups in the US, ten categories emerged:

(i) US-born White.
(ii) US-born Hispanic.
(iii) US-born Black.
(iv) US-born Asian.
(v) US-born multi-race.
(vi) Immigrant White.
(vii) Immigrant Hispanic.
(viii) Immigrant Black.
(ix) Immigrant Asian.
(x) Immigrant multi-race.

US nativity included participants’ country of birth and their self-reported length of residency in the US; five categories emerged:

(i) Born in the US/natives.
(ii) Lived in the US for < 5 years.
(iii) Lived in the US 5–14 years.
(iv) Lived in the US for 15–30 years.
(v) Lived in the US for > 30 years.

These classifications were adapted from studies by other researchers [12, 21, 22].

#### Food Security Status

Food insecurity, characterized by limited access to enough food for an active, healthy life, exists in some households in the US [23]. Food insecurity was assessed using the 18-item US Household Food Security Survey Module, a well-validated questionnaire developed by the US Department of Agriculture (USDA) to measure household food security over the prior 12 months [24]. The survey includes three questions about the food security conditions of the whole household, seven questions about the food security conditions of adults in the household, and eight questions about the food security conditions of children in the household. These questions address anxiety over insufficient household food budget or food supply, the experience of running out of food without money to obtain more, perceptions that food is inadequate in quality or quantity, and changes to food quality or reduced food intake. Using USDA-validated cut-points, the participants were categorized as food secure, marginal food security, and low or very low food security. For our analysis, we combined food secure and marginal food security into a combined “food secure” category and “low” and “very low” food security as “food insecure” [15].

#### Diet Quality

In the NHANES, dietary intake was assessed by trained interviewers using 24-h dietary recall surveys on two occasions, first day and a second day follow-up. The current study utilized the first day data to calculate diet quality (DQ). The Healthy Eating Index (HEI) 2015 was used to assess DQ scores as it was created to reflect key recommendations from the 2015–2020 US Dietary Guidelines for Americans. The HEI- 2015 consists of 13 different components based on nutrient content and food serving equivalents. Of these, nine components assess dietary adequacy:

- Total fruits.
- Whole fruits.
- Total vegetables.
- Greens and beans.
- Whole grains.
- Dairy.
- Total protein foods.
- Seafood.
- Plant proteins and fatty acids.
- Four dietary components address moderation:
  - Refined grains.
  - Sodium.
  - Added sugar.
  - Saturated fat.

For the adequacy components, higher levels of intake result in higher scores. For the moderation components, lower levels of intake result in higher scores. The scoring algorithm operates on a density basis of component amounts per 1000 cal or ratio of fatty acids (a ratio of unsaturated to saturated fatty acids). The scale of 0–10 was for seven of the components (whole grains, dairy, fatty acids, refined grains, sodium, added sugars, and saturated fats) and 0–5 for six of the components (total fruits, whole fruits, seafood, and plant proteins, total vegetables, total protein food, and greens and beans). The total HEI- 2015 score is obtained by summing the scores for all 13 components, which range from 0 to 100, with higher scores reflecting better diet quality.

#### Covariates

Covariates included in the analyses to adjust for potential confounding factors are age, gender, educational attainment (less than high school, high school graduate/GED, some college or associate degree, college graduate or higher), family poverty income ratio (PIR) calculated as the ratio of monthly family income to the federal poverty income threshold specific to the respondents’ family size), and BMI which was categorized into four levels (underweight < 18.5, normal weight 18.5–24.9, overweight 25–29.9, and obese > 30 kg/m^2^).

#### Analytic Plan

Given the complex survey design, all statistical analyses were performed using SAS version 9.4 (SAS Institute Inc., Cary, NC, USA) and STATA version 12.2 (STATA Corporation, College Station, Texas) to accommodate multiple sample weights in accordance with NHANES guidelines [25]. Sample weights accounted for differential sampling probabilities and non-response to produce accurate, unbiased national estimates during the inferential statistical analysis to establish associations.

Descriptive statistics were conducted for demographic characteristics to estimate frequencies and percentages for categorical variables, mean values, and their respective standard errors for continuous variables. For our analysis, total HEI- 2015 scores, age, and family PIR were entered as continuous variables while BMI was entered as a categorical variable. The outcome variables were food insecurity and diet quality, and logistic regression models were used to estimate associations between ethnic-immigration status and US nativity with food security status. These models compute the adjusted odds ratios (AOR) to compare the likelihood of food insecurity between groups. Linear regression models were used to compare mean total HEI- 2015 scores representing the overall diet quality across ethnic-immigration status and US nativity groups. The referent groups were US-born Whites and “born in the US/natives” for ethnic-immigration status and US nativity respectively. All adjusted models controlled for age, education, PIR, and BMI. The significance level was set at *p* < 0.05 in all the analyses.

To better visualize the multidimensional aspects of the HEI- 2015 scores, radar charts were used to relate the 13 component scores, which include both adequacy and moderation components. The mean of each component score was plotted as the percentage of the maximum score; the outer edge of the radar represents a score of 100% for that component and the center of the radar represents a score of 0% for any given component.

## Results

The study sample included 6933 adults with an average age of 48.4 years (standard error, SE ± 0.60) and an average family income poverty ratio of 3.2 (SE ± 0.05). Half of the participants (52.1%) were females, 83.3% have high school and higher certifications, and most (43.8%) were obese. By ethnic-immigration status, one-third (35.4%) were US-born Whites, 24.6% were US-born Blacks, immigrant Hispanics were 12.9%, and immigrant Asians were 9.5%. The majority of the respondents were US-born (73.8%), 2.0% had been in the United States for < 5 years, 4.8% within 5–14 years, 9.3% within 15–30 years, and 9.4% for > 30 years. A total of 62.9% participants were classified as food secure. The participants’ average total HEI score was 49.7 (SE ± 0.45). Further details are provided in Table 1.

**Table 1:** Sociodemographic characteristics of adult participants aged 18 years and older in NHANES 2017–2020 (*N* = 6933)

Variable	Mean ± SE	*n* (%)
Age	48.4 ± 0.60	
Family income poverty ratio	3.2 ± 0.05	
HEI total score	49.7 ± 0.45	
Gender		
Female		3612 (52.1)
Male		3321 (47.9)
Education		
Less than High school		1159 (16.7)
High school graduate/GED		1678 (24.2)
Some college or associate degree		2346 (33.8)
College graduate or higher		1750 (25.2)
BMI		
Underweight		98 (1.4)
Normal		1613 (23.3)
Overweight		2187 (31.5)
Obese		3035 (43.8)
Ethnic-immigration status		
US-born Whites		2455 (35.4)
US-born Hispanics		558 (8.1)
US-born Blacks		1704 (24.6)
US-born Asians		97 (1.4)
US-born multi-racial		303 (4.4)
Immigrant Whites		104 (1.5)
Immigrant Hispanics		892 (12.9)
Immigrant Blacks		130 (1.9)
Immigrant Asians		661 (9.5)
Immigrant multi-racial		29 (0.4)
US nativity		
Born in the US		5117 (73.8)
Lived in the US for < 5 years		191 (2.0)
Lived in the US for 5–14 years		331 (4.8)
Lived in the US for 15–30 years		641 (9.3)
Lived in the US for > 30 years		653 (9.4)
Food secure		4367(62.9)

Table 2 shows that those who have lived in the US for less than 5 years (AOR = 1.84, 95% CI = 1.06–3.20) and those who have lived in the US for 30 years or more (AOR = 1.62, 95% CI = 1.16–2.24) have higher odds of experiencing food insecurity compared to US-born individuals.

**Table 2:** Associations of food insecurity status and diet quality with US nativity among adult participants aged 18 years and older in NHANES 2017–2020 (*N* = 6933)

Outcome variable	Food insecurity*		Diet quality (total HEI score)**	
US nativity	**AOR**^**†**^	**95% confidence interval**	**β (mean difference)**	***P*-value**
Lived in the US for < 5 years Lived in the US for 5–14 years Lived in the US for 15–30 years Lived in the US for > 30 years	1.84 0.60 1.36 1.62	**1.06–3.20** 0.31–1.17 0.95–1.96 **1.16–2.24**	7.78 7.26 5.86 4.43	** < 0.0001** ** < 0.0001** ** < 0.0001** ** < 0.0001**

Models controlled for age, education, PIR, and BMI. NHANES analytical weights were applied during the data analyses

^**†**^AOR is adjusted odds ratio. “Born in the US” was the referent for US nativity

^*^Weighted logistic regression model

^**^Weighted linear regression model

Regarding diet quality, compared to US-born participants, those who have lived in the US for less than 5 years had the highest mean difference of the total HEI score value of 7.78 (*p* < 0.0001). Those who have lived in the US between 5 and 14 years had a mean difference of 7.26 (*p* < 0.0001), followed by those who have lived in the US for 15 to 30 years at a mean difference of 5.86 (*p* < 0.0001) and those who have lived in the US for 30 years and above at a mean difference of 4.43 (*p* < 0.0001).

Table 3 shows the associations of food insecurity and diet quality with ethnic-immigration status. US-born Asians (AOR = 0.39, 95% CI 0.17–0.90) and immigrant Whites (AOR = 0.40, 95% CI = 0.19–0.86) have lower odds of experiencing food insecurity compared to US-born Whites. However, the odds of experiencing food insecurity were about two times for US-born multi-racial group (AOR = 2.36, 95% CI 1.51–3.67) and immigrant Hispanics (AOR 2.20, 95% CI 1.55–3.12). US-born Hispanics (AOR = 1.69, 95% CI = 1.19–2.38) and Blacks (AOR = 1.511, 95% CI = 1.19–1.91) have higher odds of experiencing food insecurity compared to US-born Whites.

**Table 3:** Associations of food insecurity status and diet quality with ethnic-immigration status among adult participants aged 18 years and older in NHANES 2017–2020

Outcome variable	Food insecurity*		Diet quality (total HEI score)**	
Ethnic-immigration status	**AOR**^**†**^	**95% confidence interval**	***β* (mean difference)**	***P*-value**
US-born Hispanics	**1.69**	**1.19–2.38**	1.35	0.25
US-born Blacks	**1.51**	**1.19–1.91**	0.69	0.29
US-born Asians	**0.39**	**0.17–0.90**	**3.93**	**0.002**
US-born multi-racial	**2.36**	**1.51–3.67**	1.42	0.28
Immigrant Whites	**0.40**	**0.19–0.86**	**8.53**	**0.001**
Immigrant Hispanics	**2.20**	**1.55–3.12**	**5.76**	** < 0.0001**
Immigrant Blacks	1.17	0.50–2.71	**5.77**	** < 0.0001**
Immigrant Asians	1.23	0.75–1.83	**6.06**	** < 0.0001**
Immigrant multi-racial	0.85	0.14–5.41	0.32	0.90

Models controlled for age, education, PIR, and BMI. NHANES analytical weights were applied during the data analyses

^**†**^AOR is adjusted odds ratio. “US-born White” as was the referent for ethnic-immigration status

^*^Weighted logistic regression model

^**^Weighted linear regression model

The diet quality analyses showed that immigrant Whites had the highest Healthy Eating Index (HEI) scores (*β =* 8.53; *p* < 0.001), followed by immigrants Asians (*β =* 6.06, *p* < 0.001). Furthermore, the immigrant Blacks had a HEI score of 5.77 (*p* < 0.0001), immigrant Hispanics had a diet quality of 5.76 (*p* < 0.0001), immigrant multi-racial group had the lowest HEI score among the immigrant groups with a value of 0.32 (*p =* 0.90). Among the US-born groups, US-born Asians had the highest diet quality (*β* = 3.93 and *p =* 0.02), followed by US-born multi-racial (*β =* 1.42 and *p =* 0.28). Also, US-born Hispanics had HEI score of (*β =* 1.35 and *p* 0.25), while US-born Blacks had the lowest diet quality in the group (*β* = 0.69 and *p =* 0.29).

**Fig. 2:**
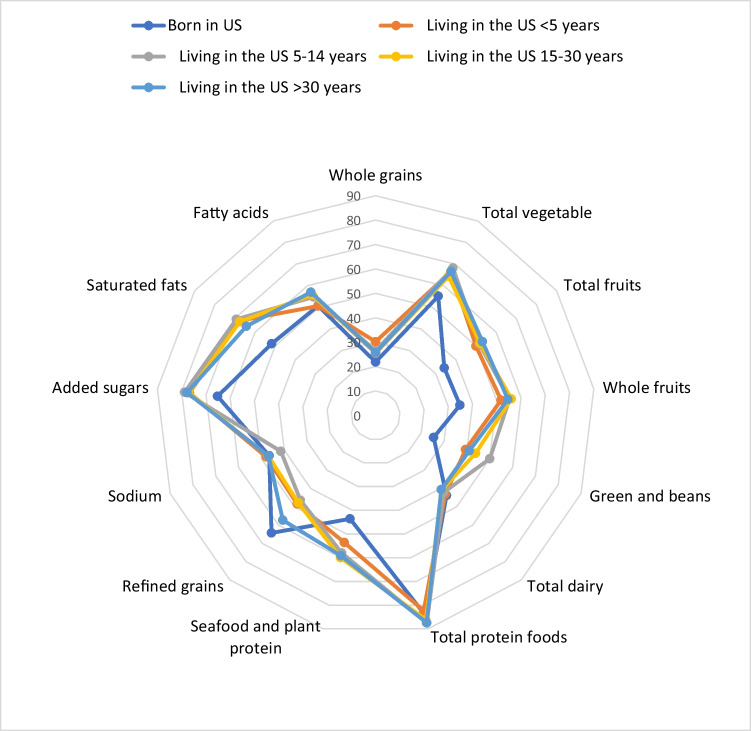
Ratios of HEI- 2015 component mean scores to maximum scores among adult participants aged 18 years and older in NHANES 2017–2020 by US nativity

As shown in Fig. 2, US-born individuals show the lowest adherence for most of the adequate HEI components, namely whole grain, total vegetables, total fruits, whole fruits, green and beans, seafood, and plant protein. The HEI scores of those who are not native to the United States were higher and comparable to these adequate HEI components. Total dairy and protein intake were comparable across all groups studied; the chart in Fig. 1 indicates closer adherence to recommended protein intake for all groups, with lower adherence for total dairy among all groups. For the moderation components, US-born participants had a lower HEI score for added sugar, saturated fat, and fatty acid indicating higher consumption. Furthermore, all groups showed lower adherence to the recommended HEI score for sodium with participants who had lived in the US for 5–14 years having the lowest HEI scores. While the HEI score for refined grain was generally low for all groups, it was higher for US-born participants, followed by participants who had lived in the US for more than 30 years. This is an indication of moderate consumption of refined grains compared to other individuals.

**Fig. 3:**
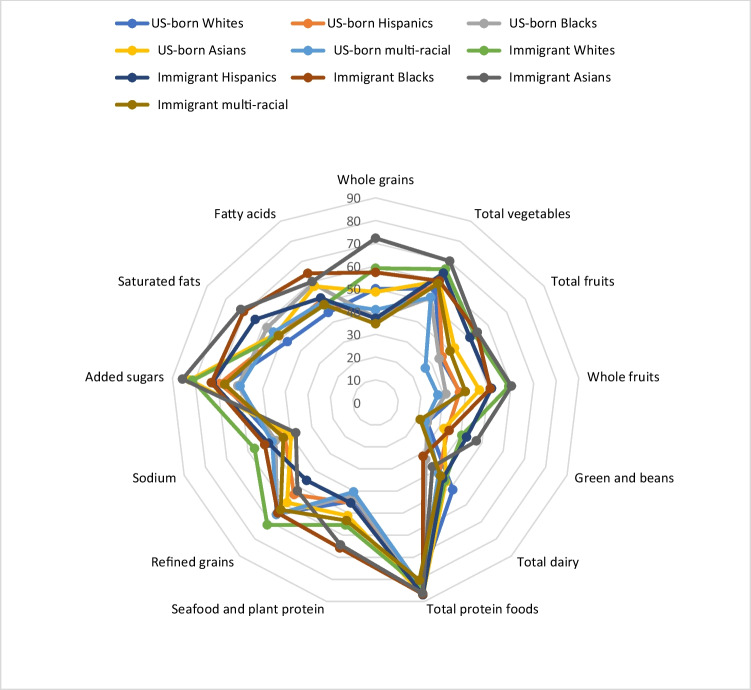
Ratios of HEI- 2015 component mean scores to maximum scores among adult participants aged 18 years and older in NHANES 2017–2020 by ethnic-immigration status

As depicted in Fig. 3, immigrant Asians and Whites consistently had higher HEI scores for adequate components, including whole grains, total vegetables, total fruits, and greens and beans. Immigrant and US-born multi-racial groups and immigrant Hispanics have the lower HEI score for whole grain. US-born Whites, Blacks, and multi-racial individuals had lower total vegetable and fruit intake, whereas greens and peas intake were low among US-born Whites, Blacks, multi-racial, and immigrant multi-racial groups. For total protein, the graph indicates a closer adherence to recommended protein intake for all groups. Immigrant Blacks, Hispanics, and US-born Blacks have the lowest adherence for total diary; US-born and immigrant Whites have the highest adherence. For whole fruit, immigrant Whites and Asians have one of the highest HEI scores followed by immigrant Blacks and Hispanics. US-born Blacks and multi-racial individuals have the lowest HEI scores for whole fruit. Immigrant Asians and Blacks have the highest HEI scores for seafood and plant proteins followed by immigrant Hispanics and US-born Asians. In contrast, US-born Whites, Hispanics, Blacks, multiracial groups, and immigrant Hispanics show lower levels of adherence to recommendations for seafood and plant protein intake.

For the moderation components, immigrant Whites, immigrant Asians, and US-born Asians had the highest HEI score for added sugar, indicating lower consumption. Conversely, US-born Blacks and multi-racial individuals have the lowest HEI score for added sugar. The HEI score for saturated fat and fatty acid was higher for immigrant Blacks and Asians compared to immigrant multi-race, US-born Whites, and multi-racial who had the lowest HEI for these components. For sodium, immigrant Whites, Blacks, and Hispanics have a higher HEI score, compared to US and immigrant Asians, who had lower HEI scores. Immigrant Whites, Blacks, multiracial, and US-born multiracial individuals have higher HEI scores for refined grains compared to other groups. In contrast, immigrant Hispanics, Asians, and US-born Hispanics have a higher intake of refined grains with lower HEI scores.

## Discussion

This study examines the differences in food insecurity risk and diet quality (using HEI score) across different lengths of residency and ethnic-immigration status. The findings show a higher likelihood of food insecurity for individuals who have lived in the US for less than 5 years and more than 30 years compared to those who were born in the US. Moreover, when considering differences across different ethnic-immigration groups, US-born Hispanics, Blacks, multi-racial groups, and immigrant Hispanics have a higher likelihood of experiencing food insecurity than US-born Whites. For dietary quality, our study demonstrated that the overall diet quality of individuals with varying lengths of residency in the US surpasses that of individuals born in the US. Among ethnic-immigration groups, immigrant Hispanics, Blacks, and US-born Asians demonstrated overall diet quality that surpassed that of US-born Whites.

Our findings on the association between food insecurity and length of residency align with a previous study, which reported that food insecurity is most prevalent among new immigrants, decreases after 5–14 years of residency in the US, and then rises again after 15 years [12]. These findings highlight the dynamic nature of food insecurity over the immigrant lifecycle, influenced by structural, economic, and social factors [26, 27]. However, this finding contrasts with another study that reported that the rates of food insecurity do not appear to decline with duration among the foreign-born, non-citizen [10]. New immigrants may experience the highest levels of food insecurity, likely due to challenges such as limited financial resources, unfamiliarity with the US food systems, language barriers, and limited access to social network support [2, 11, 12, 14, 15]. After 5 years of residency, food security status does not significantly differ from that of US-born individuals in our study. However, food insecurity increases again after more than 30 years of residency. This initial improvement may be attributed to immigrants achieving greater financial stability, accessing better employment opportunities, building support networks, or adapting to US systems and culture [28]. The subsequent rise in food insecurity could be linked to factors such as aging, health-related challenges, and economic stagnation [29, 30].

Furthermore, our study revealed that food insecurity disproportionately affected US-born Hispanics, Blacks, and multi-racial individuals, highlighting significant disparities among these groups. The observed disparity in food insecurity among US-born minority groups stems from economic challenges, including lower income levels and limited employment opportunities [13, 31]. Additionally, structural issues such as living in food deserts, systemic discrimination, and inadequate access to social safety nets exacerbate the problem. These factors are further compounded by intergenerational poverty, which fosters inequalities in wealth and education that put more households at risk of food insecurity [32] and are more likely for those immigrant categories with language barriers, unfamiliarity with food assistance programs, discrimination, dietary pattern changes, and acculturation challenges [33]. High risk to food insecurity among multi-racial groups may be due to experiences with discrimination and social exclusion [34, 35] which may affect their economic opportunities and access to food. Previous studies suggest that perceived discrimination is a significant predictor of food insecurity [36–38].

Moreover, these findings align with prior research, emphasizing the importance of intersectionality in understanding health and nutrition disparities [13, 16]. US-born individuals from minority groups may face distinct challenges compared to their immigrant counterparts, even within the same ethnic background, highlighting the complexity of these disparities [13, 16, 39]. While there are a few studies that have explicitly analyzed the combined effects of ethnic and immigrant status on nutrition outcomes, a previous cross-sectional study examined the differences in food insecurity among ethnic and racial groups by nativity and language use and found that there were significant trends in food insecurity among Hispanic adults, depending upon nativity and language use [13]. Conversely, a secondary analysis of a nationally representative dataset study reported higher food security among specific immigrants, such as those from China and India [16]. In our study, which used US-born Whites as the comparison group, we observed no significant difference in the food security status between immigrant Black, Asians, multi-racial group, and US-born White. However, US-born Asians and immigrant Whites are more food secure, and immigrant Hispanics are more food insecure than US-born Whites. These findings attest to the role of immigrant selection, which tends to favor certain groups [16]. Immigration selection is guided by certain traits, such as education, health, or economic resources, which shape who migrates and under what circumstances. Positive immigration selection may occur when immigration policies prioritize individuals with specific skills, education levels, or professional experiences to meet labor market needs [40, 41]. As a result, highly skilled immigrants who moves to a new country may be healthier, wealthier, or more skilled than their native counterparts. Individuals from Asian and European backgrounds often migrate through high-skilled pathways (including student and exchange visitors, employment-based migration), which may contribute to greater food security observed among these groups in this study. Conversely, negative immigration selection may be experienced when individuals with fewer resources or lower skills migrate, often driven by economic hardship (undocumented and humanitarian-based immigration from conflict-affected regions) [42, 43]. Africans, Caribbeans, and Latinx are likely to migrate under economic or political distress. Moreover, immigrants from marginalized ethnic groups may face systemic barriers such as language limitations, lack of credential recognition, and discrimination in the labor market. This can diminish the advantages of positive selection for some skilled immigrants. Additionally, the difficulties of migration, economic challenges or discrimination, language barriers, unfamiliarity with food assistance programs, and adjustments to new dietary patterns might limit food security among these immigrant groups. Thus, immigrant selection can shape health disparities between immigrants and the native-born population, especially when considering factors like ethnic background, immigration status, or immigration pathways. By identifying these disproportionate impacts, our findings underscore the need for targeted interventions that address the root causes of food insecurity and promote equity in food access and security across all demographics.

As far as the authors are aware, no prior research has specifically investigated variations in diet quality across the general population based on lengths of residency and ethnic-immigration status. By length of residency, our findings suggest that recent immigrants adhere more closely to dietary guidelines compared to US-born individuals and that there is a gradual decline in their diet quality over time as the duration of residence increases. This decline may be due to the adoption of less healthy eating habits, which are common in the US food environment. Over time, as immigrants integrate into US society, they adopt Western dietary habits characterized by higher consumption of processed foods, added sugars, and saturated fats, leading to lower diet quality and HEI scores [44, 45]. Moreover, evidence from the HEI- 2015 component mean score ratios presented in Fig. 2 highlights US-born individuals’ high intake of added sugar, saturated fat, and fatty acid. Meanwhile, the intake of sodium was higher for individuals who had lived in the US for 5–14 years compared to others, indicating lower adherence to dietary recommendations. Greater exposure to US food markets, including fast food and larger portions, coupled with economic and time constraints, further encourages reliance on less nutritious options, and social pressures to fit in may also result in reduced consumption of traditional foods and increased adoption of mainstream US diets [46]. Over time, limited access to traditional ingredients and cooking practices erodes cultural food traditions, contributing to the observed decline in dietary quality among long-term immigrants [47]. However, it is surprising to observe a moderate consumption of refined grain among US-born and individuals who have lived in the US for more than 30 years compared to other immigrants by the duration of their residency. Refined grains may not be a universally familiar concept to recent immigrants, as their awareness depends on cultural, dietary, globalization experiences, and educational backgrounds. Some immigrants come from regions where whole grains are traditionally consumed [45], and the idea of “refined grains” might not be explicitly recognized or emphasized in their food culture. However, exposure to refined grain products like white bread, pasta, and white rice may occur quickly after immigration, especially in countries like the US, where such products are common. Conversely, immigrants may have already been exposed to US culture through media, education, and global networks due to the influence of globalization [48]. This pre-migration exposure may have extended to their dietary habits as observed for those with higher consumption of refined grains. Moreover, our results show generally poor adherence to whole grain recommendation among all groups, though adherence appears to be slightly higher for recent immigrants. Additionally, the low intake of fruits and vegetables (total vegetables, total fruits, whole fruits, green, and peas) is a common issue across these groups, with the lowest adherence for US-born individuals. To address these dietary issues, targeted interventions that focus on increasing access to affordable, culturally appropriate whole grains, fruits, and vegetables and dietary education campaigns focused on reducing refined grains consumption could help improve adherence to dietary recommendations among all groups.

Immigrants with different lengths of residency have a higher HEI than people born in the US, indicating that they tend to comply more with dietary recommendations. This finding emphasizes the effect of cultural food habits in addition to the protection that the traditional way of eating provides, particularly in the early stages of residency, whereby people consume nutrient-rich foods like fruits, vegetables, and whole grains [49, 50]. Our findings on disparities in diet quality based on ethnic-immigrant status underscore the interaction between cultural dietary patterns (which are often shaped by their ethnicity or country of origin) and exposure to the US food environment (such as the availability, affordability, and marketing of food in the US). Asians (both the US-born and immigrant groups) have a significantly healthier overall diet compared to US-born Whites. Similarly, immigrant Whites, Hispanics, and Blacks have healthier overall diets, reflecting the influence of traditional dietary habits and cultural food preferences that persist upon arrival in the US. Literature suggests that recent immigrants often adhere more closely to traditional diets, which tend to be richer in whole foods such as vegetables, fruits, and grains, and lower in processed foods and sugars compared to the typical Western diet [44, 45]. Figure 3 further corroborates these findings by highlighting consistently higher HEI scores for immigrant Asians, Whites, and Blacks for total fruits, whole fruits, total vegetables, whole grains, greens and beans, seafood, and plant protein. Mirroring the pattern seen in food security, the improved diet observed among individuals of Asian and European backgrounds may be attributed to positive immigration selection factors explained above. Immigrant Hispanics showed moderate consumption of whole grains, total fruits, whole fruits, and total vegetables. Additionally, immigrant Whites and Blacks have better adherence to refined grain, sodium, and added sugar recommendations, while immigrant Hispanics have lower adherence to the recommendations for refined grain [51]. This difference may be attributed to cultural food practices that emphasize whole grains, lower sodium intake, and less reliance on unprocessed foods and ingredients among immigrant Whites and Blacks [52]. The findings underscore the importance of culturally appropriate tailored interventions to curb the systemic barriers and enhance the diet quality of immigrants [16].

Moreover, immigrant Blacks, immigrant Asians, and US-born Asians have some of the lowest fatty acid and saturated fat intake. Traditional diets in some Asian and African cultures may emphasize cooking methods such as steaming or boiling rather than frying, which can reduce fat intake [53]. Immigrant groups may also retain these dietary habits longer before adapting to more fat-rich Western diets. These dietary patterns highlight the protective role of cultural food traditions in moderating fat consumption. It is noteworthy that both US-born and immigrant Blacks had one of the lowest adherences to dairy products. In addition to cultural dietary patterns, this may be influenced by high rates of lactose intolerance among Black populations, which can discourage the consumption of dairy products [54]. US-born Whites have lower adherence to recommendations for saturated fats and fatty acids; this is not surprising given the US food environment, where there is often increased consumption of processed and fast foods, major sources of saturated fats. These findings underscore the need for culturally tailored nutrition interventions to address dietary imbalances while respecting cultural food practices.

One of the strengths of this research is that it was based on NHANES, a nationally representative dataset with standardized procedures to ensure comparability and reliability across demographic and dietary assessments. Such a rich data source enhances the generalizability of our findings regarding acculturation, and ethnicity—a proxy for cultural identity, food security, and dietary quality. Acculturation may often be viewed as a one-way process where immigrants gradually adopt the host culture. However, by incorporating ethnicity, our study acknowledges the possibility of individuals maintaining aspects of their heritage culture. Additionally, our study examines individual-level changes while recognizing the influence of systemic barriers such as discrimination, socioeconomic inequality, and restrictive immigration policies on the acculturation process. Moreover, the categorization approach used in constructing intersectionality acknowledges the diverse immigrant experiences that shape the acculturation process.

However, the data relied on self-reported dietary assessment, subject to potential biases such as recall and social desirability biases. The study recognizes the potential for selection bias from filtering the dataset based on specific criteria. However, this process ensures the sample aligns with the study’s objectives with no missing outcome, demographics, and socio-economic status data. Missing data for other characteristics were handled using listwise deletion, the default in SAS. Furthermore, the cross-sectional design of the NHANES may limits the possibility of drawing causal inferences about the relationships between acculturation, ethnicity, and diet quality. Further research could employ a longitudinal study design to capture more nuanced dietary changes and the evolving nature of acculturation while utilizing a stratified sampling method to ensure a comprehensive representation of the nativity and ethnicity of the targeted groups. Despite the limitations, the study provided invaluable insights into how social-cultural environmental factors including acculturation and cultural identity influences diet quality and opined the need for tailored public health programs.

## Conclusion

This study shows that recent immigrants and certain ethnic groups may face initial challenges with food security. However, as the length of residency increases, food security tends to improve indicating adaptation over time. Despite this improvement, longer residency may lead to shifts toward less healthy dietary patterns, possibly due to increased exposure to processed and convenience foods. Furthermore, food insecurity may increase again with extended residency. Additionally, the study highlights how cultural food traditions and dietary education interact with the US food systems and available food choices in the US, leading to variations in diet quality among different ethnic and immigrant groups. Moreover, this study underscores the importance of examining how overlapping identities (intersectionality) can shape people’s experiences of food insecurity and diet quality. These interconnected identities, including ethnic background and immigrant status, can create unique challenges and contribute to health disparities, necessitating a more nuanced approach to understanding and addressing nutrition and health challenges.

### Implication for Practice and Programs

Equity-focused food access programs are essential for reducing food insecurity and improving the diet quality of US-born and immigrant populations, especially Blacks, Hispanics, and multi-racial groups. Additionally, food security can be enhanced by increasing access to healthy foods in food deserts, expanding subsidies for nutritious foods, and strengthening programs such as the Supplemental Nutrition Assistance Program (SNAP). Creating supportive environments for sustainable healthy eating requires a multi-faceted approach, with collaboration from policymakers, communities, and related industries.

These findings highlight the importance of culturally tailored nutritional interventions that not only address dietary imbalances but also respect and integrate cultural food practices to promote healthier eating habits. Additionally, promoting greater cultural sensitivity and incorporating diverse food traditions into mainstream nutrition education can help US natives adopt healthier eating habits, drawing from the positive aspects of immigrant dietary patterns. Each group has valuable dietary practices to share, and nutrition interventions can facilitate the exchange of these practices, encouraging cross-cultural learning that benefits all populations. By emphasizing the strengths of each group’s food traditions, interventions can foster mutual learning and improve overall dietary patterns across diverse communities. Moreover, future research could examine the sustainability of culturally responsive interventions over time and their activeness in reducing dietary disparities and improving food security.

## Data Availability

The dataset is attached as a supplemental material.

## Code Availability

The codes used to generate the findings may be made available upon request.
